# Results of a pilot study on the involvement of bilateral inferior frontal gyri in emotional prosody perception: an rTMS study

**DOI:** 10.1186/1471-2202-11-93

**Published:** 2010-08-10

**Authors:** Marjolijn Hoekert, Guy Vingerhoets, André Aleman

**Affiliations:** 1 BCN-Neuroimaging Center, University Medical Center Groningen, University of Groningen, Antonius Deusinglaan 2, PO Box 196, 9700 AD Groningen, the Netherlands; 2 Laboratory for Neuropsychology, Department of Internal Medicine, Section Neurology, University Ghent, 4K3, De Pintelaan 185, B-9000 Ghent, Belgium

## Abstract

**Background:**

The right hemisphere may play an important role in paralinguistic features such as the emotional melody in speech. The extent of this involvement however is unclear. Imaging studies have shown involvement of both left and right inferior frontal gyri in emotional prosody perception. The present pilot study examined whether these brain areas are critically involved in the processing of emotional prosody and of semantics in 9 healthy subjects. Repetitive transcranial magnetic stimulation was used with a coil centred over left and right inferior frontal gyri, as localized by neuronavigation based on the subject's MRI. A sham condition was included. An online-TMS approach was applied; an emotional language task was completed during stimulation. This computerized task consisted of sentences pronounced by actors. In the semantics condition an emotion (fear, anger or neutral) was expressed in the content pronounced with a neutral intonation. In the prosody condition the emotion was expressed in the intonation, while the content was neutral.

**Results:**

Reaction times on the emotional prosody task condition were significantly longer after rTMS over both the right and the left inferior frontal gyrus as compared to sham stimulation and after controlling for learning effects associated with order of condition. When taking all emotions together, there was no difference in effect on reaction times between the right and left stimulation. For the emotion Fear, reaction times were significantly longer after stimulating the left inferior frontal gyrus as compared to the right inferior frontal gyrus. Reaction times in the semantics task condition were not significantly different between the three TMS conditions.

**Conclusions:**

The data indicate a critical involvement of both the right and the left inferior frontal gyrus in emotional prosody perception. The findings of this pilot study need replication. Future studies should include more subjects and examine whether the left and right inferior frontal gyrus play a differential role and complement each other, e.g. in the integrated processing of linguistic and prosodic aspects of speech, respectively.

## Background

In auditory language processing, distinct brain areas serve different aspects of language. Language has been attributed to the left hemisphere since Broca (1861) and Wernicke (1874). Their studies showed that articulate speech and verbal comprehension are disrupted by left but not right hemisphere lesions [1]. Emotional prosody, a paralinguistic feature of language, is characterized by intonation, loudness and stress placement in speech. The emotional prosody of spoken language may convey crucial information about the emotional state of the speaker. Not only *what *is said but also *how *it is said gives significant information about the speaker's true communicative intent and is therefore crucial for proficient social interaction [2]. Studies examining the neural substrate of emotional prosody perception have revealed a network including bilateral regions in superior and middle temporal gyri and orbital and inferior frontal regions. Some studies have also implicated sub cortical structures such as the amygdala [3,4] and the basal ganglia [5,6]. Lesion and imaging studies have suggested that processing of affective prosodic information may be differentially lateralized when compared to linguistic, semantic processing. Whereas (in right-handers) the left hemisphere seems to be specialized for semantic and syntactic components of speech, the right hemisphere appears to be dominant in non-lexical components, such as affective prosody and gestural signs in communication [7,8]. Lesion and imaging studies show, however, discrepant data to the extent of "right lateralization" of emotional prosody processing. A study that directly compared emotional prosody discrimination against discrimination of emotional semantics revealed right lateralized activity during detection of emotional prosody and left lateralized activity as a response to emotional semantics [9]. A number of imaging studies have also shown right lateralized activity during the perception of emotional prosody [3,9-14]. Other imaging studies on emotional prosody perception have however shown neural responses in both right and left hemispheres [15-22]. Deficits in emotional prosody perception have also been found after left hemisphere lesions [23,24]. To further define the exact neural substrate of emotional prosody perception and the extent of its lateralization, we used transcranial magnetic stimulation (TMS), a brain mapping technique that allows causal inferences between neural activity and performance at a behavioural level [25,26]. A recent TMS study provided evidence of right-hemisphere involvement in emotional prosody discrimination [27]. Increased reaction times were observed after 12 min of 1 Hz TMS (90% of the motor threshold) over the right fronto-parietal operculum relative to a left-hemisphere sham condition. This effect was specific for emotional prosody discrimination and was not found for discriminating emotional semantics. Detection of withdrawal emotions and not of approach emotions in prosody was delayed significantly by TMS, in accordance with accounts of the neural implementation of approach and withdrawal systems [28].

The region of interest, the right fronto-parietal operculum, in the study of van Rijn et al., was based on a lesion study from Adolphs et al. [23,27]. The present experiment was a sequel and extension to the study of van Rijn [27]. The target regions of our study were not based on lesion studies, however, but on imaging studies revealing an association with bilateral inferior frontal gyri [14,21]. We used on-line TMS in order to test the following hypotheses. First, both left and right inferior frontal gyri are critically involved in emotional prosody perception. Second, emotional semantics and emotional prosody can be dissociated at a neuro-anatomical level. Finally, there is a difference in lateralization between withdrawal (fear) and approach (anger) emotions.

## Methods

### Participants

Ten subjects (6 females, 4 males), students from the University of Groningen aged between 18 and 26 years (mean 21.8, s.d. 2.6 years) participated in the study. Right-handedness was confirmed using the Edinburgh Handedness Inventory by using the scoring method: (Total left - total right)/(total left + total right) * 100. Results < -40 = left-handed, between -40 and + 40 = ambidextruous and results > + 40 = right-handed. They had at least a high school level of understanding of Dutch (comprehension and reading). Participants were screened for TMS exclusion criteria [29] and MRI exclusion criteria. None had a (family) history of psychiatric or neurological problems or implants. They were given extensive written and oral explanation of the procedures and signed an informed consent. The experiment was conducted in accordance to the Declaration of Helsinki and local ethics committee approval (University Medical Centre Groningen).

### Experimental setup

**Figure 1:**
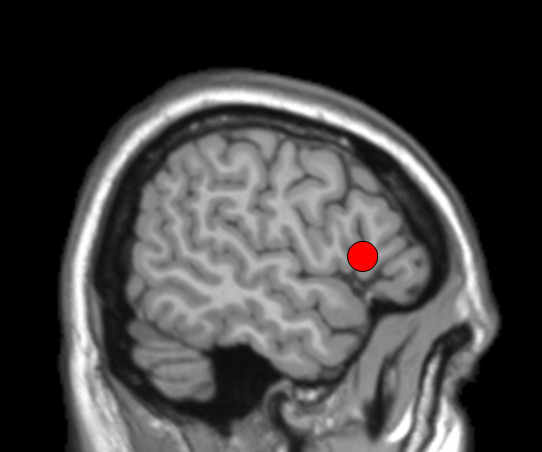
**Region of interest, the inferior frontal gyrus, drawn in MRIcro**.

Structural scanning was done on a Philips Intera 3 T MR-system at the BCN Neuroimaging Center, Groningen. The regions of interest were drawn in MRIcro a few days before the TMS experiment by the primary investigator and checked by another researcher until the two reached consensus, see Figure 1. Number of voxels per region of interest was ± 120, 1 to 2 cm, conform the size of the region TMS affects [30]. The bilateral inferior frontal gyrus was defined as BA 45/46 conform Ethofer et.al. [21]. For the TMS procedure, subjects were seated in a comfortable chair, in front of a computer screen and a keyboard with coloured response keys. A neural navigator (NeNa) was used to reliably localize the desired stimulation areas, of the participant [31]. This frameless stereotaxy device allows using a subject's structural MRI scan to navigate a TMS coil to the proper location on the skull. At the beginning of each TMS session the left and right inferior frontal gyri were marked on a tightly fit rubber head cap. For TMS, we used a MagStim Rapid magnetic stimulator (MagStim Co, Whitland, UK) with a figure of eight coil with a diameter of 70 mm for each loop. Then, individual motor thresholds of the left hemisphere were determined using the thumb (abductor pollicis brevis) movement procedure [32]. Stimulation intensity was set at 90% of the motor threshold, mean stimulation intensity was 52.1% (s.d. 3.1). In one subject the stimulation intensity had to be decreased from 59 to 54 because of an uncomfortable feeling while stimulating the right inferior frontal gyrus. In another subject we could not succeed in determining the motor threshold, for this subject the stimulation intensity was set at 50 of maximum output. Maximum output of the TMS machine is 2.5 Tesla. The coil was tightly maintained in a constant position with a special developed arm. The orientation of the coil was set with the handle pointing downwards, making an angle of 90° with the midline of the head.

### Emotional language task

The emotional language task consisted of two conditions, prosody and semantics. These were matched in design. In the affective prosody condition subjects had to attend to the intonation of the voice and ignore the neutral content. The sentences of neutral content are pronounced in an emotional (anxious, angry) or neutral tone of voice by two professional actors, a male and a female voice, to control for individual and/or gender differences in affective prosody. All sentences were Dutch. Examples of the sentences with neutral content are, "The old car drives through the streets of the capital" and "Jan has been going to the hairdresser". In the semantics condition, subjects had to attend to the emotional (anxious, angry) or neutral content, and ignore the neutral intonation. Examples of sentences with an emotional content are, "Desperately he threw the glass from the table" and "The face stared with a pale face". The sentences were selected from two different validated prosody tests [33,34]. The digitized stimuli for both conditions were all of approximately equal length (about seven words) and presented through two computer sound boxes (duration varying from 1560 to 3050 msec). The task was developed and presented using Eprime software [35]. During listening, the emotions to be discriminated, anger, fear or neutral, were presented on the computer screen. The visual presentation of the answer choices was included to aid subjects as to which categories they were to choose from. Without the visual presentation, subjects would have to memorize the different categories, and the task would have a stronger working memory component. As soon as participants identified the emotion expressed in the tone of voice, they were required to use the index finger of their right hand to make a "fear" response on a keypad, the middle finger to make an "angry" response or the thumb for a "neutral" response. Speed and accuracy were stressed.

### TMS Procedure

At the beginning of each TMS session, subjects completed the positive and negative affect schedule (PANAS), to assess their current affective state [36] (Dutch translation from Peeters et al., [37], validated by Boon and Peeters, [38]). Before the TMS stimulation started, participants performed a practice task for one of the two conditions (semantics or prosody) to get used to the task. An on-line task design was employed, i.e. participants completed the tasks during the TMS stimulation. The design used was described in a recent study on covert speech arrest induced by rTMS, done by Aziz-Zadeh et al. [39]. The experiment ran in three blocks of the same length, corresponding to the three TMS conditions: the two stimulated scalp positions and a sham condition; the areas of stimulation were the left and right inferior frontal gyrus (IFG). A third condition concerned the sham control condition, which was over the right IFG. In the sham condition we used a placebo coil, of which the manifestation and the clicking sound are similar to a real coil. During (sham) stimulation of each area 48 stimuli, consisting of 24 trials of each task condition (semantics and prosody), resulting in 8 trials per emotion (anger, fear or neutral), were presented. Order of conditions and TMS stimulated scalp positions were counterbalanced across subjects. RTMS during the auditorally presented stimuli consisted of a train of 12 pulses at 5 Hz. The train was delivered starting 200 msec prior to stimulus presentation. A new stimulus was presented every 10 sec. The time interval between the TMS trains was 7600 msec. The TMS blocks were separated by at least 30 min in order to minimize the possibility of carry-over effects [40]. Participants were instructed to respond as quickly and as accurately as possible by pressing one of three coloured keys on a keypad. The entire procedure took 160 min at maximum in one session.

### Statistical Analysis

Statistical analyses were performed using Statistical Package for the Social Sciences 14.0.0 (2005). Accuracy, described as percentage correct, and reaction times for detection of emotion in semantics and prosody were chosen as dependent variables. As participants performed highly accurately (prosody condition 90% on average (s.d. 4.7), semantics condition 92% (s.d. 7.9)), only reaction times for correct trials were included in the analyses. In the design used in this study, every experimental trial was accompanied either by real TMS or sham stimulation. A 2 × 3 × 3 repeated measures ANOVA with task condition (prosody, semantics), TMS condition (left inferior frontal gyrus, right inferior frontal gyrus or sham) and emotion (fear, anger or neutral) as within subject factors and 'order of TMS conditions' as between subjects variable was used to test the effect of TMS on distinct location on the two tasks for the three emotions. Given that we used a within-subjects design in which subjects received different TMS (or sham) conditions in one session, we had to take into account learning effects as a result of repeated testing. Learning effects due to repeated testing have been shown in a variety of neuropsychological tests [41] and can be so large that they might obscure experimental effects. Therefore, the variable 'order of TMS conditions' was included as a between subjects factor in the repeated measures ANOVA, to control for the order of TMS conditions. One subject scored a percentage correct of 38% in the sham condition of the semantics task; we excluded her results from the analysis because this percentage is close to chance level. Results from nine subjects were included in the analysis. Results with p-values < .05 were regarded as significant.

## Results

### Mood and speed-accuracy trade off

One-factor repeated measures ANOVA, with TMS condition as a factor, revealed that there was no relation between the TMS conditions and the PANAS positive and negative scales. Therefore, it is unlikely that differences in reaction times and accuracy scores between the TMS conditions can be accounted for by changes in mood. There was no speed-accuracy trade off effect: no correlation was found between reaction times and accuracy scores. Results also did not reveal learning effects; accuracy scores and reaction times were equal per subject over the conditions. All effects were tested at a significance level of p < .05.

### Accuracy

With regard to accuracy, no significant differences between the TMS conditions or any interaction effects were found. Mean percentage correct in the semantics condition was 92% (s.d. 7.9) and in the prosodic condition, 90% (s.d. 4.7).

### Reaction times

For mean reaction times, a main effect of task was found, *F *(1, 8) = 19. 4, *p *< .05. Longer reaction times were found in the semantics condition than in the prosody condition, mean reaction times were 2412 msec (s.d. 629 msec) and 2063 msec (s.d. 443 msec) respectively. Therefore both tasks were analysed separately.

**Figure 2:**
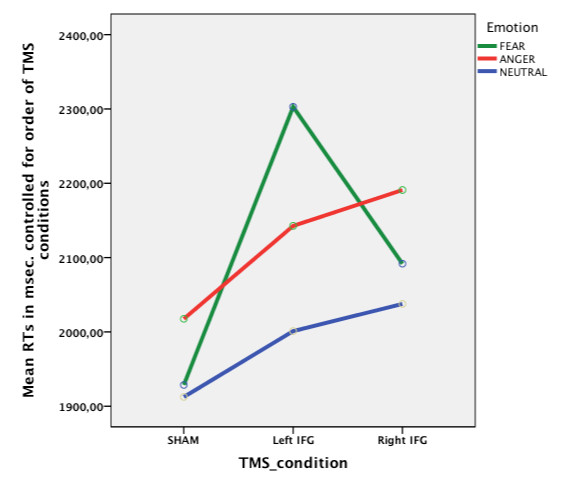
**Reaction times on prosody task for the three TMS conditions**. Mean reaction times per TMS condition on the emotional prosody task corrected for order of TMS conditions. Separate lines represent the three emotions, fear, anger and neutral.

Analysis on the prosody task, with mean reaction times for the three TMS conditions (sham, left inferior frontal gyrus and right inferior frontal gyrus) as within subjects' variable and order of TMS conditions as between subjects' variable, revealed a main effect of TMS condition, *F *(2,8) = 13.4, *p *< .01. If all other variables are ignored, reaction times were different between the three TMS conditions for the prosodic task condition. Contrasts revealed that reaction times were longer after rTMS over both left and right inferior frontal gyrus as compared to sham, *F *(1, 4) = 13.3, *p *< .05 and *F *(1, 4) = 15.7, *p *< .05 respectively. There was also no difference between TMS over left inferior frontal gyrus and TMS over right inferior frontal gyrus, *F *(1, 4) = 5.3, *p *= .08 when comparing reaction times on the prosody task. There was however, also an interaction between TMS condition and order of TMS conditions, *F *(8, 8) = 20.2, *p *< .001. This indicates that differences in reaction times between the TMS conditions in the prosodic task condition (left IFG, right IFG and sham) depend on the order of TMS conditions. Although this interaction was found, the results shown in Figure 2 and the main effect of TMS condition show that reaction times in the prosody condition were longer after both left and right TMS, after correction for order of TMS conditions. The estimated means show that reaction times after real TMS are longer, if the sham condition is included as second or third condition.

To test the third hypothesis: there is a difference in lateralization between withdrawal (fear) and approach (anger) emotions, Emotion was added as a within subjects' variable. No difference between the three emotions was found, *p *= .21 and no interaction between emotion and condition, when taking into account the order of TMS condition, *p *= .32. Therefore, the three emotions should be analysed together. When looking at the separate lines for the emotions in Figure 2, however, there seems to be a difference between the TMS conditions, for the emotion fear. When analyzing this emotion separately, there is indeed a significant difference between the three TMS conditions, after correcting for the order of TMS conditions, *F *(8, 8) = 3.48, *p *< .05. Contrasts revealed significant longer reaction times after stimulating the left inferior frontal gyrus than after stimulating the right inferior frontal gyrus in detecting the emotion fear from prosody, *F *(4, 4) = 9.48, *p *< .05). For the emotion anger, no effect of condition was found, *p *= 0.13. The difference represented in Figure 2 between the lines representing fear and anger is not significant, no interaction was found between TMS condition and emotion, *p *=.32. Although the relation looks quadratic, contrasts revealed no difference in reaction times between fear and anger depending on the TMS condition, *p *= .12.

Analysis on the reaction times in the semantics task condition, with TMS condition and Emotion as within subjects' variables and order of TMS conditions as between subjects' variable revealed no significant differences between TMS conditions and no interaction effects.

## Discussion

This pilot study was designed to test three hypotheses: 1) Both left and right inferior frontal gyri are critically involved in emotional prosody perception. 2) Emotional semantics and emotional prosody can be dissociated at a neuro-anatomical level. 3) There is a difference in lateralization between withdrawal (fear) and approach (anger) emotions. The first hypothesis was supported by the results of this pilot study. Participants were slower in correctly classifying emotion from prosody during TMS over both left and right inferior frontal gyrus as compared to a sham condition. This indicates that both the left and the right inferior frontal gyrus are crucially involved in emotional prosody perception. Our findings thus support a recent theoretical framework, proposed by Schirmer and Kotz [42]. These authors argue that both left and right are involved in the processing of emotional prosody, albeit with different roles. More specifically, the right inferior frontal gyrus would be involved in evaluative judgments, while the left inferior frontal gyrus would sub serve the integration with other co-occurring processes [42]. The target regions of the present study were based on an imaging study of Ethofer et al. [21]. This study has lent support for a cooperation of the left and right inferior frontal gyrus in affective prosody perception, by testing their effective connectivity [21]. These authors performed a connectivity analysis which indicated a flow of information along parallel projections from the right posterior superior temporal cortex to the bilateral inferior frontal cortices [21]. In our study stimuli in the prosody condition consisted of sentences with a neutral semantic content. Both evaluative judgment of the prosodic intonation and integration with co-occurring processes, like the integration with emotional semantic meaning from earlier experiences, were needed to be able to choose the correct emotion. Inhibiting one of these two areas is enough to deteriorate reaction times on this task. Apparently, imaging studies have already shown the involvement of both left and right frontal areas in emotional prosody perception. As far as we know, this is the first study with TMS showing the crucial involvement of left and right inferior frontal gyrus in emotional prosody perception, which gives a stronger test of causal involvement. This finding leaves open a possible rightward asymmetrical activation at a temporal level, as has been found in imaging studies [9,11,12,21]. No effects were found on accuracy measures, this is as expected, because virtual lesions induced by TMS generally manifest in reaction times rather than in percentages correct [43]. Our data did not show significant involvement of the left inferior frontal gyrus in emotional semantics. This is in contradiction with other studies examining the neuro-anatomical substrate of processing emotional information from semantics [9,27,44]. This might be explained by the high inter-subject variability and the sensitivity of the semantics condition. It could have been that the sentences used as stimuli and the number of emotions (the two emotions anger and fear and neutral) in the semantics condition was too easy. Furthermore, the absence of effect in the semantic condition can be due to lack of power. The second hypothesis of a differential involvement of the right hemisphere in emotional prosody and of the left hemisphere in emotional semantics that was found in an imaging study [9] was not supported by our data. Our study could not dissociate the processing of emotional semantics and emotional prosody at a neuro-anatomical level. The third hypothesis on a difference in lateralization between withdrawal (fear) and approach (anger) emotions could also not be supported by our data. Performances on both emotions in the prosody condition deteriorated after rTMS over left and over right inferior frontal gyrus. Interestingly, the separate analysis of reaction times for the detection of the emotion fear from prosody revealed significantly longer reaction times after TMS over the left inferior frontal gyrus as compared to stimulation over the right inferior frontal gyrus. This finding is in contradiction with accounts of the neural implementation of approach and withdrawal systems [28] and with the findings from a recent TMS study of van Rijn et al. [27]. In that study however, a different brain area was targeted (fronto-parietal operculum) and that study lacked a left TMS condition. Furthermore, more emotions were included, not only anger and fear, as in our study, but also happiness and sadness. This could have made the task more sensitive and did result in more trials after splitting in withdrawal and approach emotions. Some limitations of present study should be noted. The first concern is the use of a placebo coil. Although its manifestation and the clicking sound are similar to a real coil, it does not give the same sensations as real TMS. That is, sensations are absent for the placebo coil. We cannot exclude general TMS induced effects on attention as a consequence of the sensations. Other sham conditions however, also have drawbacks. It has been shown, that tilting the coil by 45 to 90 degrees, which is the most frequently used sham condition, might still affect brain activity [45]. Another option is to include an active control condition, a region that has never been related to the function of interest. This method can also not be regarded as an unaffected baseline, because it may cause the general TMS induced effects on attention. Inclusion of temporal cortex regions in the design could give a stronger test of lateralization of emotional information processing in both [21] semantics and in prosody.

## Conclusions

In summary, our pilot data lend evidence from TMS for a crucial involvement of both right and left inferior frontal gyrus in emotional prosody perception consistent with earlier fMRI findings [21]. The findings of this pilot study need replication. Future research should investigate whether the left and right inferior frontal gyrus play a differential role and complement each other, e.g. in the integrated processing of linguistic and prosodic aspects of speech, respectively [42]. The number of subjects should be higher in future studies.

## Authors' contributions

Authors MH and AA designed the study, MH wrote the article and did the statistical analyses, both thoroughly discussed with AA. GV helped with the interpretation of the data, checked the manuscript for completeness, and gave good suggestions for improvement of the text. All authors contributed to and have approved the final manuscript.

## References

[B1] Ross ED Thompson RD Yenkosky J Lateralization of affective prosody in brain and the callosal integration of hemispheric language functions Brain and language 1997 56 27 54 10.1006/brln.1997.1731 8994697

[B2] Mitchell RL Crow TJ Right hemisphere language functions and schizophrenia: the forgotten hemisphere? Brain 2005 128 963 978 10.1093/brain/awh466 15743870

[B3] Phillips ML Young AW Scott SK Calder AJ Andrew C Giampietro V Williams SC Bullmore ET Brammer M Gray JA Neural responses to facial and vocal expressions of fear and disgust Proceedings of the Royal Society of London Series B 1998 265 1809 1817 10.1098/rspb.1998.0506 9802236 PMC1689379

[B4] Sander D Grandjean D Pourtois G Schwartz S Seghier ML Scherer KR Vuilleumier P Emotion and attention interactions in social cognition: Brain regions involved in processing anger prosody NeuroImage 2005 28 848 858 10.1016/j.neuroimage.2005.06.023 16055351

[B5] Pell MD Leonard CL Processing emotional tone from speech in Parkinson's disease: a role for the basal ganglia Cognitive, affective & behavioral neuroscience 2003 3 275 288 10.3758/cabn.3.4.275 15040548

[B6] Cancelliere AE Kertesz A Lesion localization in acquired deficits of emotional expression and comprehension Brain and cognition 1990 13 133 147 10.1016/0278-2626(90)90046-Q 1697174

[B7] Ross ED Orbelo DM Cartwright J Hansel S Burgard M Testa JA Buck R Affective-prosodic deficits in schizophrenia: profiles of patients with brain damage and comparison with relation to schizophrenic symptoms Journal of Neurology, Neurosurgery, and Psychiatry 2001 70 597 604 10.1136/jnnp.70.5.597 11309452 PMC1737346

[B8] Ross ED The aprosodias Behavioral neurology and neuropsychology 2003 McGraw Hill. New York

[B9] Mitchell RL Elliott R Barry M Cruttenden A Woodruff PW The neural response to emotional prosody, as revealed by functional magnetic resonance imaging Neuropsychologia 2003 41 1410 1421 10.1016/S0028-3932(03)00017-4 12757912

[B10] George MS Parekh PI Rosinsky N Ketter TA Kimbrell TA Heilman KM Herscovitch P Post RM Understanding emotional prosody activates right hemisphere regions Archives of neurology 1996 53 665 670 8929174 10.1001/archneur.1996.00550070103017

[B11] Wildgruber D Riecker A Hertrich I Erb M Grodd W Ethofer T Ackermann H Identification of emotional intonation evaluated by fMRI NeuroImage 2005 24 1233 1241 10.1016/j.neuroimage.2004.10.034 15670701

[B12] Beaucousin V Lacheret A Turbelin M Morel M Mazoyer B Tzourio-Mazoyer N FMRI study of emotional speech comprehension Cerebral cortex 2007 17 339 352 10.1093/cercor/bhj151 16525130

[B13] Imaizumi S Mori K Kiritani S Kawashima R Sugiura M Fukuda H Itoh K Kato T Nakamura A Hatano K Vocal identification of speaker and emotion activates different brain regions Neuroreport 1997 8 2809 2812 10.1097/00001756-199708180-00031 9295122

[B14] Buchanan TW Lutz K Mirzazade S Specht K Shah NJ Zilles K Jancke L Recognition of emotional prosody and verbal components of spoken language: an fMRI study Cognitive Brain Research 2000 9 227 238 10.1016/S0926-6410(99)00060-9 10808134

[B15] Morris JS Scott SK Dolan RJ Saying it with feeling: neural responses to emotional vocalizations Neuropsychologia 1999 37 1155 1163 10.1016/S0028-3932(99)00015-9 10509837

[B16] Johnstone T van Reekum CM Oakes TR Davidson RJ The voice of emotion: an FMRI study of neural responses to angry and happy vocal expressions Soc Cogn Affect Neurosci 2006 1 242 249 10.1093/scan/nsl027 17607327 PMC1905858

[B17] Wildgruber D Pihan H Ackermann H Erb M Grodd W Dynamic brain activation during processing of emotional intonation: influence of acoustic parameters, emotional valence, and sex NeuroImage 2002 15 856 869 10.1006/nimg.2001.0998 11906226

[B18] Kotz SA Meyer M Alter K Besson M von Cramon DY Friederici AD On the lateralization of emotional prosody: An event-related functional MR investigation Brain and language 2003 86 366 376 10.1016/S0093-934X(02)00532-1 12972367

[B19] Meyer M Alter K Friederici AD Lohmann G von Cramon DY FMRI reveals brain regions mediating slow prosodic modulations in spoken sentences Hum Brain Mapp 2002 17 73 88 10.1002/hbm.10042 12353242 PMC6871847

[B20] Ethofer T Kreifelts B Wiethoff S Wolf J Grodd W Vuilleumier P Wildgruber D Differential Influences of Emotion, Task, and Novelty on Brain Regions Underlying the Processing of Speech Melody Journal of cognitive neuroscience 2008 21 7 1255 68 10.1162/jocn.2009.21099 18752404

[B21] Ethofer T Anders S Erb M Herbert C Wiethoff S Kissler J Grodd W Wildgruber D Cerebral pathways in processing of affective prosody: A dynamic causal modeling study NeuroImage 2006 30 580 587 10.1016/j.neuroimage.2005.09.059 16275138

[B22] Wildgruber D Hertrich I Riecker A Erb M Anders S Grodd W Ackermann H Distinct Frontal Regions Subserve Evaluation of Linguistic and Emotional Aspects of Speech Intonation Cerebral cortex 2004 14 1384 1389 10.1093/cercor/bhh099 15217896

[B23] Adolphs R Damasio H Tranel D Neural systems for recognition of emotional prosody: a 3-D lesion study Emotion 2002 2 23 51 10.1037/1528-3542.2.1.23 12899365

[B24] Hornak J Bramham J Rolls ET Morris RG O'Doherty J Bullock PR Polkey CE Changes in emotion after circumscribed surgical lesions of the orbitofrontal and cingulate cortices Brain 2003 126 1691 1712 10.1093/brain/awg168 12805109

[B25] Pascual-Leone A Walsh V Rothwell JC Transcranial magnetic stimulation in cognitive neuroscience--virtual lesion, chronometry, and functional connectivity Current opinion in neurobiology 2000 10 232 237 10.1016/S0959-4388(00)00081-7 10753803

[B26] Kucharska-Pietura K Phillips ML Gernand W David AS Perception of emotions from faces and voices following unilateral brain damage Neuropsychologia 2003 41 1082 1090 10.1016/S0028-3932(02)00294-4 12667543

[B27] van Rijn S Aleman A van Diessen E Berckmoes C Vingerhoets G Kahn RS What is said or how it is said makes a difference: role of the right fronto-parietal operculum in emotional prosody as revealed by repetitive TMS European Journal of Neuroscience 2005 21 3195 3200 10.1111/j.1460-9568.2005.04130.x 15978028

[B28] d'Alfonso AAL van Honk J Hermans E Postma A de Haan EHF Laterality effects in selective attention to threat after repetitive transcranial magnetic stimulation at the prefrontal cortex in female subjects Neuroscience Letters 2000 280 195 198 10.1016/S0304-3940(00)00781-3 10675794

[B29] Wassermann EM Risk and Safety of repetitive transcranial magnetic stimulation: report and siggested guidelines from the International Workshop on the safety of repetitive transcranial magnetic stimulation, June 5-7,1996 1998 10.1016/s0168-5597(97)00096-8 9474057

[B30] Walsh V Cowey A Transcranial magnetic stimulation and cognitive neuroscience Nature Reviews Neuroscience 2000 1 73 79 10.1038/35036239 11252771

[B31] Neggers SFW Langerak TR Schutter DJLG Mandl RCW Ramsey NF Lemmens PJJ Postma A A stereotactic method for image-guided transcranial magnetic stimulation validated with fMRI and motor-evoked potentials NeuroImage 2004 21 1805 1817 10.1016/j.neuroimage.2003.12.006 15050601

[B32] Pridmore S Fernandes FJA Nahas Z Liberatos C George MS Motor threshold in transcranial magnetic stimulation: A comparison of a neurophysiological method and a visualization of movement method The journal of ECT 1998 14 25 10.1097/00124509-199803000-00004 9661090

[B33] Vroomen J Collier R Mozziconacci S Duration and intonation in emotional speech 577 580

[B34] Vingerhoets G Berckmoes C Stroobant N Cerebral hemodynamics during discrimination of prosodic and semantic emotion in speech studied by transcranial Doppler ultrasonography Neuropsychology 2003 17 93 99 10.1037/0894-4105.17.1.93 12597077

[B35] Schneider W Eschman A Zuccolotto A E-Prime User's Guide 2002 Pittsburgh, Psychology Software Tools Inc

[B36] Watson D Clark LA Tellegen A Development and validation of brief measures of positive and negative affect: the PANAS scales Journal of Personality and Social Psychology 1988 54 1063 1070 10.1037/0022-3514.54.6.1063 3397865

[B37] Peeters F Ponds R Vermeeren M Affectiviteit en zelfbeoordeling van depressie en angst Tijdschrift voor psychiatrie 1996 38 240 250

[B38] Boon MTG Peeters FPML Affectieve dimensies bij depressie en angst Tijdschrift voor psychiatrie 1999 41 109 113

[B39] Aziz-Zadeh L Cattaneo L Rochat M Rizzolatti G Covert speech arrest induced by rTMS over both motor and nonmotor left hemisphere frontal sites Journal of cognitive neuroscience 2005 17 928 938 10.1162/0898929054021157 15969910

[B40] Kosslyn SM Pascual-Leone A Felician O Camposano S Keenan JP Thompson WL Ganis G Sukel KE Alpert NM The role of area 17 in visual imagery: convergent evidence from PET and rTMS Science 1999 284 167 170 10.1126/science.284.5411.167 10102821

[B41] Wilson BA Watson PC Baddeley AD Emslie H Evans JJ Improvement or simply practice? The effects of twenty repeated assessments on people with and without brain injury Journal of the International Neuropsychological Society 2000 6 469 479 10.1017/S1355617700644053 10902416

[B42] Schirmer A Kotz S Beyond the right hemisphere: brain mechanisms mediating vocal emotional processing Trends in cognitive sciences 2006 10 24 30 10.1016/j.tics.2005.11.009 16321562

[B43] Wassermann E Epstein CM Ziemann U Oxford handbook of transcranial stimulation 2008 Oxford: Oxford University Press

[B44] Baum SR Pell MD The neural bases of prosody: Insights from lesion studies and neuroimaging Aphasiology 1999 13 581 608 10.1080/026870399401984

[B45] Loo C Taylor J Gandevia S McDarmont B Mitchell P Sachdev P Transcranial magnetic stimulation (TMS) in controlled treatment studies: are some "sham" forms active? Biological psychiatry 2000 47 325 331 10.1016/S0006-3223(99)00285-1 10686267

